# Improved self-training-based distant label denoising method for cybersecurity entity extractions

**DOI:** 10.1371/journal.pone.0315479

**Published:** 2024-12-17

**Authors:** Ke Zhang, Yunpeng Wang, Ou Li, Sirui Hao, Junjiang He, Xiaolong Lan, Jinneng Yang, Yang Ye

**Affiliations:** 1 Nuclear Power Institute of China, Chengdu, China; 2 Smart Rongcheng Operation Center in Xindu District, Chengdu, China; 3 School of Cyber Science and Engineering, Sichuan University, Chengdu, China; King Saud University, SAUDI ARABIA

## Abstract

The task of named entity recognition (NER) plays a crucial role in extracting cybersecurity-related information. Existing approaches for cybersecurity entity extraction predominantly rely on manual labelling data, resulting in labour-intensive processes due to the lack of a cybersecurity-specific corpus. In this paper, we propose an improved self-training-based distant label denoising method for cybersecurity entity extraction. Firstly, we create two domain dictionaries of cybersecurity. Then, an algorithm that combines reverse maximum matching and part-of-speech tagging restrictions is proposed, for generating distant labels for the cybersecurity domain corpus. Lastly, we propose a high-confidence text selection method and an improved self-training algorithm that incorporates a teacher-student model and weight update constraints, for exploring the true labels of low-confidence text using a model trained on high-confidence text, thereby reducing the noise in the distant annotation data. Experimental results demonstrate that the cybersecurity distantly-labelled data we obtained is of high quality. Additionally, the proposed constrained self-training algorithm effectively improves the F1 score of several state-of-the-art NER models on this dataset, yielding a 3.5% improvement for the Vendor class and a 3.35% improvement for the Product class.

## Introduction

An abundance of security data exists in the form of unstructured natural language text or semi-structured vulnerability databases. These databases include the National Vulnerability Database(NVD) [1], Common Platform Enumeration (CPE) [2], and Exploit Database (Exploit-DB) [3]. When researchers perform cybersecurity-related tasks, such as intrusion detection, threat intelligence base building, etc., they need to perform Named Entity Recognition (NER) on these large amounts of cybersecurity data for structured security data analytics [4–6]. Therefore, the field of NER has attracted significant attention from researchers.

Gasmi et al. [7] proposed the use of LSTM-CRF for extracting network security entities, achieving good results by leveraging the memory effect of LSTM. Huang et al. [8] first introduced BiLSTM-CRF for NER tasks, which is one of the most representative deep learning-based NER models. Chui et al. [9] proposed the use of an LSTM-CNN model for NER, which utilizes CNN instead of CRF as a post-processing layer and can extract character-level features. Ma et al. [10] combined BiLSTM, CNN, and CRF, introducing BiLSTM-CNN-CRF, which was one of the best-performing NER models before the emergence of pre-training models. Tikhomirov et al. [11] introduced BERT into the field of network security NER, achieving better results compared to other traditional models.

In summary, many researchers have conducted studies on NER, primarily focusing on improvements in model architecture. However, NER still faces challenges such as a lack of high-quality annotated data and high annotation costs, especially in domains like cybersecurity that require expert knowledge. On one hand, training NER models with good performance requires a large amount of high-quality annotated data for supervised training. On the other hand, obtaining high-quality annotated data is difficult, particularly in domains like cybersecurity that are highly specialized and lack publicly available datasets with high confidence levels. Notably, NER models trained on specific datasets often underperform when applied to different ones [12]. Therefore, transferring NER models from one domain to another is not an effective approach.

To address these problems, in this paper, we propose an improved self-training-based distant label denoising method for cybersecurity entity extraction. Firstly, we build two cybersecurity domain dictionaries as remote knowledge bases. Secondly, we propose an algorithm called **R**everse **M**aximum Matching combined with **P**OS condition (referred to as **RMP**) to generate distant labels, then we utilize a high-confidence text selection criterion to identify and select high-confidence texts, train an initial NER model on these texts. Lastly, we propose a framework called **I**mproved **S**elf-**T**raining (referred to as **IST**), which incorporates the teacher-student model and weight update constraints. This framework iteratively predicts and explores the true labels of low-confidence texts, updating the weights. By doing so, it reduces the impact of noisy labels and gradually improves the performance of the model. The experimental results demonstrate the effectiveness of our method. The main contributions of this paper are as follows:

- We propose a matching algorithm that combines Reverse Maximum Matching (RMM) with POS conditions. This method differs from traditional forward dictionary matching approaches and achieves higher precision and recall rates in distant supervision.
- We propose an improved self-training algorithm that combines a teacher-student model with weight update condition constraints. This algorithm can effectively reduce the impact of noisy labels.
- We evaluate the quality of the obtained distant labels and the effectiveness of the improved self-training algorithm on NER models. The experimental results demonstrate that the cybersecurity distant data we obtained has good quality, and the constrained self-training algorithm can effectively improve the performance of several state-of-the-art NER models on this dataset.

## Related work

Cybersecurity entity extraction is a use of NER tasks in the field of cybersecurity. As an important task of NLP, NER has always been given attention by researchers and many related algorithms have been proposed. Most previous studies mainly focused on a fully supervised approach, which requires a well-annotated corpus. These studies usually consider the neural network models to fit label distribution. Huang et al. [8] presented the BiLSTM-CRF model, which is one of the most representative deep learning-based NER models. They considered named entity recognition as a sequence annotation task. Ma et al. [10] presented BiLSTM-CNNS-CRF, which uses Convolutional Neural Networks(CNN) to extract features from the characters of words, splice them with GloVe word embeddings, and finally feed them into BiLSTM-CRF for training.

In order to reduce human labour and quickly label a large amount of data, distant supervision is used to automatically label training data with a domain-specific dictionary or public knowledge base. Mayhew et al. [13] proposed Constrained Binary Learning (CBL), a self-training algorithm that iteratively identifies true negative labels in NER to mitigate noise impact and improve the effectiveness of NER models.

Liang et al. [14] combined this technology with the pre-training model, and put forward the BOND model, which was applied to the long-distance annotated text obtained based on knowledge base matching, and used the way of early stopping to prevent the fitting noise. Meng et al. [15] presented the RoSTER model, which removes noise labels by introducing a noise robustness loss function based on generalized cross-entropy and makes the model more stable by adopting model integration and other methods.

Compared to other domains, entity extraction in cybersecurity is less studied and faces many problems. Gasmi et al. [7] proposed LSTM-CRF to extract entities in cybersecurity, and also performed feature engineering without expert knowledge. Satyapanich et al. [16] expanded the entity categories and utilized BERT and Word2Vec pre-trained word embeddings in combination with a BiLSTM model to extract entity information from network security articles. Georgescu et al. [17] designed an automated diagnostic system for the Internet of Things (IoT) cybersecurity environment. They used a semi-supervised method Watson Knowledge Studio tool to train the NER model for heterogeneous IoT data. Evangelatos et al. [18] employed fine-tuning of state-of-the-art transformer-based models for NER in Cyber Threat Intelligence. Alam et al. [19] proposed CyNER, a user-friendly Python library that provides access to transformer-based NER models pre-trained on high-fidelity security events along with a heuristic model for extracting threat indicators. Zhen et al. [20] proposed RoBERTa-wwm-RDCNN-CRF, an end-to-end NER model for Chinese Cyber Threat Intelligence that utilizes a robustly optimized pre-trained RoBERTa with whole word masking, leading to significant improvements in entity recognition performance. Hu et al. [21] introduced JCLB, an innovative model that integrates Contrastive Learning with a Belief Rule Base to enhance NER accuracy in the cybersecurity domain.

In summary, while numerous studies have focused on improving Named Entity Recognition (NER) through advancements in model architecture, significant challenges remain, especially in specialized domains like cybersecurity. The complexity and diversity of cybersecurity texts make it difficult for traditional NER methods to accurately extract security-related entities. High-performing NER models typically require large volumes of high-quality annotated data for supervised training, but the lack of a domain-specific corpus poses a significant obstacle. Moreover, the scarcity of publicly available, reliable datasets makes obtaining high-quality annotations even more challenging. As a result, NER models trained on one dataset often struggle to generalize effectively to others. Additionally, many existing models struggle with handling and detecting noise in the data. To address these issues, we propose an enhanced self-training-based distant label denoising method specifically tailored for cybersecurity entity extraction.

## Proposed method

**Fig 1:**
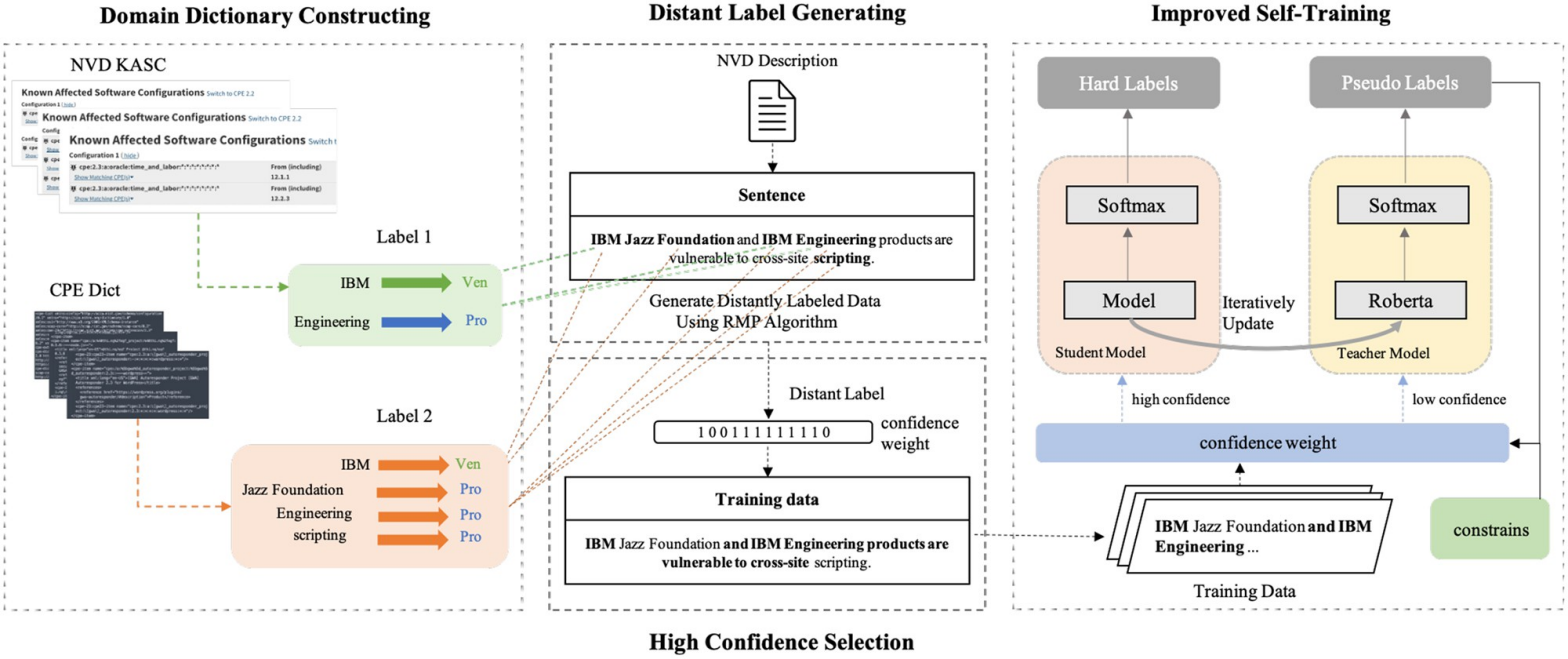
The entire framework.

To address the issue of manual annotation dependency in cybersecurity NER, we propose an improved self-training-based distant label denoising method for cybersecurity entity extraction, which includes domain dictionary construction, distant label generation, high-confidence text selection, and an improved self-training approach. The overall framework of the method is illustrated in Fig 1.

### Domain dictionary construction

**Fig 2:**
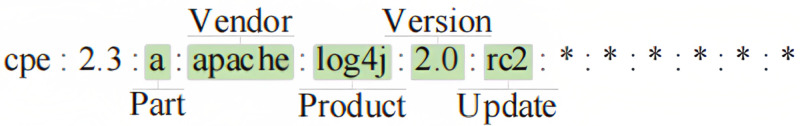
Example of CPE URI version 2.3.

This section elucidates the construction of domain dictionary. We use the NVD’s Description text as the target corpus, and the CPE list as the external knowledge base. As indicated in Fig 2, a CPE URI includes multiple fields. We choose “vendor” and “product” as the distant labels to be annotated for two reasons: (1) These two labels are important in the field of cybersecurity as they help in quickly identifying potential targets under threat. (2) These two labels are more commonly found in NVD texts and are easier to extract, whereas other labels may not always be present.

There are two types of CPE dictionaries that we can use: a list of CPE URIs provided by the NVD for related vulnerabilities, known as Known Affected Software Configurations, and a comprehensive official CPE dictionary. In the subsequent description, we will refer to the distant labels generated using the first dictionary as “Label 1” and the ones generated using the second dictionary as “Label 2”. It should be noted that Label 1 is more precise, while Label 2 provides a broader coverage of vulnerability.

### Distant label generation

Distant labels in an unlabeled corpus are typically obtained by matching the entity with those in the external knowledge base. In this work, we employ the reverse maximum matching algorithm to identify entities from the sentences that exist in a pre-defined dictionary. After that, the identified entities undergo POS condition filtering to assign them the appropriate class labels. Our RMP algorithm consists of two main parts: reverse maximum matching and POS condition filtering.

**Algorithm 1:** RMM Algorithm.

**Input:** An unlabeled text ***S* = {*x*_1_…*x*_*n*_}**;

external knowledge base ***D***;

the maximum length of entity ***k***.

**Output:** Distant label $Y^{DIS}=\left\{y_{1}^{DIS}...y_{n}^{DIS}\right\}$.

**1 foreach** *i* ∈ [1, …, *n*] **do**

**2**  //Initialize the distant labels as “O”;

**3**  $\left\{y_{i}^{DIS}{=}^{'}O^{'}\right\}$

**4 end**

**5** *i* ← *n*;

**6 while** *i* > 1 **do**

**7**  *j*_*max*_ ← min{*k*, *i* − 1};

**8**  *t* ← 1;

**9**  **for** *j* ← *j*_*max*_, …, 1 **do**

**10**   *ent* ← {*x*_*i*−*j*_…*x*_*i*_};

**11**   **if** *ent* ∈ *D* **then**

**12**    $\left\{y_{u}^{DIS}{=}^{'}Label^{'}|u\in \left[i-j,...,i\right]\right\}$;

**13**   **break**;

**14:**  **end**

**15**  //update the position of *i*;

**16**  *i* ← *i* − *t*

**17: end**

#### Reverse maximum matching

The Reverse Maximum Matching (RMM) algorithm are based on the greedy approach of maximum matching, where the analyzed string is matched with the entries in a dictionary according to certain strategies, the principle is to maximize the length of individual words or entities whenever possible. If a string is found in the dictionary, it is considered a successful match. For example, the algorithm can successfully match a specific entity like “windows_10” rather than a broader equivalent like “windows”, which can be more accurate. The algorithm is not only used for word segmentation but can also be applied to sequence labelling tasks [22–25]. The advantages of RMM include its fast speed and high accuracy, as it does not generate strange words. However, it may introduce noisy labels and struggle with handling out-of-vocabulary words. Due to its limited scalability, it is suitable for domain-specific NER tasks. Therefore, we have chosen to use them as our distant labelling approach.

**Algorithm 2:** RMP Algorithm.

**Input:** CPE list *L*_*cpe*_;

text *S* = {*x*_1_…*x*_*n*_};

POS tag $Y^{POS}=\left\{y_{1}^{POS}...y_{n}^{POS}\right\}$;

the maximum length of entity *k*.

**Output:** Distant label $Y^{DIS}=\left\{y_{1}^{DIS}...y_{n}^{DIS}\right\}$.

**1** //Initialize the dictionary;

**2** *D*_*ven*_ ← Set(), *D*_*pro*_ ← Set();

**3 for** *l* ∈ *L*_*cpe*_ **do**

**4**  *D*_*ven*_ ← The string between the third colon and the fourth colon in *l*;

**5**  *D*_*pro*_ ← The string between the fourth colon and the fifth colon in *l*;

**6 end**

**7** //Initialize the distant labels as “O”;

**8** $i\leftarrow n,\{y_{i}^{DIS}{=}^{'}O^{'}|i\in \left[1,...,n\right]\}$ ;

**9** **while** *i* ≤ *n* **do**

**10**  *j*_*max*_ ← min{*k*, *i* − 1}, *t* ← 1;

**11**  **for** *j* ← *j*_*max*_, …, 1 **do**

**12**   *hasNN* ← *False*;

**13**   *ent* ← {*x*_*i*−*j*_…*x*_*i*_};

**14**   **if** {$y_{u}^{POS}\in Set_{NN}|u\in \left[i,...,i+j\right]$} **then**

**15**    *hasNN* ← *True*;

**16**   **if** *ent* ∈ *D*_*ven*_ and *hasNN* = *True* **then**

**17**    $\left\{y_{u}^{DIS}{=}^{'}Vendor^{'}|u\in \left[i,...,i+j\right]\right\}$;

**18**    *t* ← *j*;

**19**    **break**;

**20**   **if** *ent* ∈ *D*_*pro*_ and *hasNN* = *True* **then**

**21**    $\left\{y_{u}^{DIS}{=}^{'}Product^{'}|u\in \left[i,...,i+j\right]\right\}$;

**22**    *t* ← *j*;

**23**    **break**;

**24**  **end**

**25**  //update the position of *i*;

**26**  *i* ← *i* − *t*

**27 end**

The overall flow of the RMM algorithm is presented in Algorithm 1. RMM algorithm has several advantages: (1) Higher efficiency: Since keywords and phrases in most languages tend to appear at the end of sentences, the RMM algorithm can quickly find matches, thereby improving search efficiency. (2) For longer target strings, the RMM algorithm is generally faster than the forward traversing. This is because the RMM algorithm starts matching from the end, allowing for quicker exclusion of non-matching parts and reducing the search scope.

#### POS condition filtering

To improve the accuracy of matching, we have introduced POS as a filtering criterion to determine whether to assign labels based on the corresponding POS tags of the text. We have set two filtering conditions: (1) Only words tagged as noun POS will be assigned labels, specifically NN, NNS, NNP, and NNPS. Otherwise, the label will be set as “O”. (2) If a word is followed by a numeral POS, specifically CD, the word will be assigned the label “Product”.

The reason for setting the first condition is evident because both Vendor and Product types are nouns. If a word with a different POS is matched, it indicates that the word is not the desired type in the current context. The reason for setting the second condition is that sometimes Vendor and Product names can be the same, but if followed by a numeral, it generally indicates a product version in cybersecurity texts. Therefore, it is considered as a Product. With these two simple filtering conditions, we can obtain more accurate distant labels.

#### Overall algorithm

We summarize the entire process of distant labelling as shown in Algorithm 2. Lines 1-8: Generating the remote dictionary knowledge base. Line 10: Initializing remote labels as “O”. Line 12: Truncating words starting from the maximum length. Lines 13-25: Performing distant labelling. Line 27: Updating the starting position.

### Noise elimination through improved self-training algorithm

In section, we presented a distant labelling method that aids in building a cybersecurity corpus. Nevertheless, distant supervision can result in some data being improperly labelled, thus creating noise. To mitigate this issue, we introduced an improved self-training learning framework designed to negate the effects of such noisy data. In this section, we provide a detailed description of the improved self-training method we propose. Step 1: We train an initial NER model using high-confidence texts. Step 2: We use the NER model to predict pseudo-labels for the low-confidence texts. Step 3: Based on the predicted labels and constraint conditions, we update the weights. The updated weights are then used to select high-confidence texts for the next round of training. Step 4: We iterate this training process until a pre-defined threshold is reached.

#### High confidence text selection

The first step of our method is to define high-confidence text, which will be used to train the initial NER model. Subsequent self-training iterations will be performed based on this initial model. We adhered to the following criteria while selecting high-confidence texts:

1. Words labelled by Label 1 that are not classified as “O” are selected as it has high confidence.
2. Words concurrently labelled as “O” by Label 1 and Label 2 are considered to have a high confidence label of “O”.

The logic behind the selection of high-confidence text is that Label 1 is more precise, indicating entities that are more likely to align with the true labels. On the other hand, Label 2 is more comprehensive, so if both Label 1 and Label 2 classify a token as a non-entity, there is a high probability that it is indeed a non-entity.

#### NER training

In general, NER models are trained as sequence labelling models. Specifically, for a sequence *S* = [*x*_1_, …, *x*_*n*_] and its corresponding labels *L* = [*y*_1_, …, *y*_*n*_], a NER model with parameters *θ* outputs class probabilities after applying the softmax function. The most commonly used loss function in NER is cross-entropy (CE):

$$\begin{array}{c} L_{\text{CE}}=-\sum_{i=1}^{n}\;logf_{i,y_{i}}\left(S;\theta \right), \end{array}$$ (1)

where $f_{i,y_{j}}\left(S;\theta \right)$ represents the probability (i.e., the softmax outputs) that the output class predicted by the model for a token *x*_*i*_ will be *y*_*j*_. The meanings of some formula symbols used in this paper are described in Table 1.

**Table 1:** Variable symbol of NER task.

Variable symbol	Meaning
*S*	natural language text passages
*x*_*i*_	the i-th token in the text
*L*	set of category labels corresponding to the text
*y*_*i*_	category labels corresponding to *x*_*i*_
*θ*	parameters of NER model
*f*(⋅, *θ*)	NER model
*f*_*i*_(*S*;*θ*)	model prediction on *x*_*i*_, soft label
$f_{i,y_{i}}\left(S;\theta \right)$	the probability that the model predicts the output class to be *y*_*i*_ on *x*_*i*_

In order to adapt the NER model to the algorithm and data proposed in this paper, we expand Eq 1, and provide a weighted loss function demonstrated in Eq 2. Here, *w*_*i*_ denotes the weight of the training data. If *w*_*i*_ = 1, it indicates that the data instance is from high-confidence text and should be included in the loss computation during training. On the other hand, if *w*_*i*_ = 0, it signifies that the data instance is from low-confidence text and should not contribute to the loss computation.

$$\begin{array}{c} L_{\text{IST}}=-\sum_{i=1}^{n}\;w_{i}logf_{i,y_{i}}\left(S;\theta \right), \end{array}$$ (2)

#### Constraints formulas

The basic idea of the IST algorithm is to first train an initial NER model on high-confidence text. Then, this model is used to generate soft-label predictions for the low-confidence text. After applying certain constraint conditions, some low-confidence text instances are selected and promoted to high-confidence text. This iterative training process continues until a stop condition is met. Here, the objective function for the constraint selection process is defined as follows:

$$\begin{array}{c} \max_{c,y}\sum_{i=0}^{\left|D_{U}\right|}\;c_{i}\sum_{j=0}^{\left|C\right|}\;p_{i,j}y_{i,j}, \end{array}$$ (3)

where *D*_*u*_ represents the set of low-confidence text instances, and *c*_*i*_ takes values 0 or 1, indicating whether the i-th token will be updated to high-confidence. *C* represents the number of classes, *p*_*i*,*j*_ represents the predicted probability of the i-th token in the j-th class, *y*_*i*,*j*_ takes values 0 or 1, indicating whether the i-th token is classified as the j-th class. The objective of this function is to select the low-confidence text instances with the highest predicted probabilities, i.e., the ones with the highest confidence.

$$\begin{array}{c} \sum_{j=0}^{\left|C\right|}\;y_{j}=1 \end{array}$$ (4)

$$\begin{array}{c} \sum_{i=0}^{\left|D_{U}\right|}\;c_{i}=C_{update} \end{array}$$ (5)

$$\begin{array}{c} \forall j\neq 0,\text{e}-\delta \leq \frac{\sum_{i}^{D_{U}}y_{i,j}}{\left|\text{L}\right|}\leq e+\delta \end{array}$$ (6)

The constraints of the IST algorithm are represented by three equations, as shown in Eqs 4, 5 and 6. In these equations, *C*_*update*_ represents the summation of all *c*_*i*_ values, while *L* represents the total number of labels. *C*_*update*_ directly determines the number of data instances that participate in the next iteration. There are two ways to obtain *C*_*update*_: one is to use a threshold value, as shown in Eq 7, where the count is calculated for values greater than *t*; the other is to use a random proportion, as shown in Eq 8.

$$\begin{array}{c} C_{update}={\left|D_{U}\right|}_{p_{i,j}>t} \end{array}$$ (7)

$$\begin{array}{c} C_{update}=r\left|D_{U}\right| \end{array}$$ (8)

Where r is a value between 0% and 100%.

Eq 6 represents the entity ratio constraint. This constraint is based on the observation made by Augenstein et al. [26] in their research that well-annotated datasets typically have an entity ratio of around 0.09±0.05. Therefore, this constraint is used to limit the entity label ratio obtained during the training process. If the ratio exceeds the highest limit, the low-confidence labels with relatively lower probabilities are put aside and no longer considered as high-confidence labels.

**Algorithm 3:** IST Algorithm.

**Input:** Pretrained model $f(\cdot ,\theta_{model}^{\left(0\right)})$;

high-confidence text $D_{L}$;

low-confidence text $D_{U}$;

stopping condition *C*_*stop*_;

weight update constraint *C*_*update*_.

**Output:** Final student model *f*(⋅, *θ*_*stu*_).

**1** //Initialize weight and model;

**2** The $D_{L}$’s text weight $W_{L}\leftarrow 1$, the $D_{U}$’s text weight $W_{U}\leftarrow 0$;

**3** **for** *t*_1_ ∈ [1, …, *T*_1_] **do**

**4**  $\theta_{model}^{\left(t_{1}\right)}\leftarrow$ Train on $D_{L}$;

**5 end**

**6** //teacher-student framework;

**7** $\theta_{tea}\leftarrow \theta_{model}^{\left(T_{1}\right)}$ , $\theta_{stu}^{\left(0\right)}\leftarrow \theta_{model}^{\left(T_{1}\right)}$;

**8** **while** Does not meet the stopping condition *C*_*stop*_ **do**

**9**  //pseudo-label $\tilde{y}$;

**10**  $\tilde{y}\leftarrow$ Use *f*(⋅, *θ*_*tea*_) to predict the label of $D_{U}$;

**11**  Update $W_{U}$ based on the constraint *C*_*update*_, and use the hard label *y* as the label for the data corresponding to $D_{U}$;

**12**  **for** *t*_2_ ∈ [1, …, *T*_2_] **do**

**13**   $\theta_{stu}^{\left(t_{2}\right)}\leftarrow$ Train on $D_{L}$ and $D_{U}$;

**14**  **end**

**15**  $\theta_{tea}\leftarrow \theta_{stu}^{\left(t_{2}\right)},\theta_{stu}^{\left(0\right)}\leftarrow \theta_{stu}^{\left(t_{2}\right)}$;

**16 end**

#### Improved self-training algorithm

Drawing inspiration from the CBL framework proposed by Mayhew et al. [13], we integrated a constraint method with a self-training framework based on the teacher-student model [27], enabling the iterative learning of the model parameter *θ*. The principal process is depicted in Algorithm 3. Lines 1-5: Initialize the weights for high-confidence and low-confidence text. Train the initial NER model using high-confidence text. Line 6: Initialize the teacher and student model parameters with the parameters of the initial model. Lines 10-11: Use the teacher model to predict soft labels for the low-confidence text. Update the weights based on the condition for weight update using the soft labels. Lines 12-14: Train the student model using the hard labels corresponding to the updated data. Line 15: Assign the trained student model parameters to the teacher and student models for the next iteration.

The training process continues iteratively until a stop condition is met. In this specific implementation, the stop condition is defined as the point where the number of occurrences of the two types of entities no longer significantly increases. At this point, the final trained student model is considered as the output model.

## Experiment

### Dataset

We randomly downloaded 934 CVE vulnerability information from NIST [1] and manually annotated them based on tokens in IO format for later evaluation. We divided the dataset into training and testing sets in an 8:2 ratio. The specific quantities of sentences, tokens, and entities are shown in Table 2.

**Table 2:** Dataset statistics with the number of sequences/tokens/entities of train/test dataset.

Dataset	Class	Sequence	Token	Entity
Train	Vendor/Product	747	32870	764/2630
Test	Vendor/Product	187	8057	185/655

Table 3 shows the partial annotation data for vulnerability number CVE-2021-1527. The first column is the natural language text that uses NLTK for word segmentation; the second column is the part-of-speech tag obtained using NLTK; the third column is the remote tag obtained using the current dictionary; the fourth column is the manually annotated real tag; the fifth column gives the vulnerability number to which the natural language text belongs.

**Table 3:** Example of distant labels.

text	POS	distant label	true label	CVE ID
A	DT	O	O	CVE-2021-1527
vulnerability	NN	O	O	CVE-2021-1527
in	IN	O	O	CVE-2021-1527
Cisco	NNP	B-Vendor	B-Vendor	CVE-2021-1527
Webex	NNP	B-Product	B-Product	CVE-2021-1527
Player	NNP	I-Product	I-Product	CVE-2021-1527
for	IN	O	O	CVE-2021-1527
Windows	NNP	O	B-Product	CVE-2021-1527
and	CC	O	O	CVE-2021-1527
macOS	NNP	O	B-Product	CVE-2021-1527
…	…	…	…	…

### Compared methods

We compared several state-of-the-art NER models in terms of their performance on distant supervision and full supervision. Full supervision involved training the models using a ground truth training set, while distant supervision involved comparing the performance of Label 1 and our proposed IST algorithm. All methods were evaluated on the test set.

- **FMM**: We compare the Forward Maximum Matching (FMM) algorithm [28] with the RMM algorithm. The FMM algorithm is similar to the RMM algorithm, but it scans the text from left to right. This method is commonly used for dictionary-based matching.
- **BiLSTM-CRF**: Classic NER model [8]. BiLSTM (Bi-directional LSTM) is an improvement over LSTM as it not only considers the historical information of words but also incorporates future information, resulting in better word vector representations. The CRF layer is to serve as a post-processing component, effectively constraining the model’s output to produce correct class labels.
- **LSTM-CNN**: [9] Instead of using CRF, it uses CNN as the post-processing layer. In this context, CNN is employed to extract character-level features for each word.
- **BiLSTM-CNN-CRF**: [10] One of the state-of-art NER models before the pre-trained language model, consisting of BiLSTM, CNN and CRF.
- **RoBERTa**: [29] RoBERTa (Robustly Optimized BERT Pretraining Approach) is an improved model based on BERT. It incorporates several modifications and enhancements to further enhance performance.
- **RoBERTa-wwm-RDCNN-CRF**: [20] This state-of-the-art NER model integrates a residual dilated convolutional neural network (RDCNN) with a conditional random field (CRF), enhancing entity recognition performance in Chinese Cyber Threat Intelligence.

### Experiment settings and metrics

#### Experiment settings

We set the output dimension of word2vec vectors to 100, BERT output vectors to 768, and RoBERTa output vectors to 768. The LSTM model utilizes 256 LSTM cells, while the BiLSTM model employs 128 BiLSTM cells. The CNN model consists of 5 parallel 1-dimensional CNN layers, each with 256 filters. Eq 5 utilizes the *C*_*update*_ determined by Eq 8, with *r* set to 0.2. The training rounds for high-confidence text *T*_1_ are set to 2, and the stopping condition for self-training *C*_*stop*_ is when there are no newly selected high-confidence texts satisfying the constraint condition. The ratio of our training set to test set was 8:2.

#### Metrics

For each experiment, we evaluate the performance using precision, recall, and F1 score. A higher precision indicates that the model’s predictions are more reliable, while a higher recall means that the model can better capture the actual positive instances. The F1 score combines precision and recall, making it a useful metric for overall quality assessment. These metrics provide a comprehensive understanding of the model’s performance in different aspects.

*Precision*. Precision is the ratio of correctly predicted positive observations to the total predicted positives. It reflects the accuracy of the model when it predicts a positive class. The formula is as follows:

$$\begin{array}{c} \text{Precision}=\frac{TP}{TP+FP} \end{array}$$

where TP (True Positive) represents the number of true positives, and FP (False Positive) represents the number of false positives.

*Recall*. Recall is the ratio of correctly predicted positive observations to all observations in the actual class. It reflects the model’s ability to capture all positive samples. The formula is as follows:

$$\begin{array}{c} \text{Recall}=\frac{TP}{TP+FN} \end{array}$$

where FN (False Negative) represents the number of false negatives.

*F1 score*. The F1 score is the harmonic mean of precision and recall, used to provide a balanced evaluation of the model’s precision and recall capabilities. The formula is as follows:

$$\begin{array}{c} \text{F1}\;\text{Score}=2\times \frac{\text{Precision}\times \text{Recall}}{\text{Precision}+\text{Recall}} \end{array}$$

The F1 score balances precision and recall, making it particularly useful for datasets with imbalanced classes.

### Distant label construction experiment results

We conduct experimental evaluations on the distantly generated labels using the RMP algorithm to showcase the quality of our distant labels, as shown in Table 4.

**Table 4:** Distant label quality evaluation (in %).

Method	Class	Label 1			Label 2		
		Pre.	Rec.	F1	Pre.	Rec.	F1
**fmp**	vendor	82.46	92.82	87.33	**76.34**	88.44	81.94
	product	79.01	90.18	84.23	51.96	96.98	67.67
	micro	79.88	91.04	85.14	55.69	95.06	70.23
	macro	80.74	91.50	85.78	64.15	92.71	74.81
**rmp**	vendor	**83.56**	**94.52**	**88.70**	76.33	**88.80**	**82.09**
	product	**79.21**	**90.28**	**84.38**	**51.99**	**97.05**	**67.71**
	micro	**80.18**	**91.24**	**85.36**	**55.73**	**95.19**	**70.30**
	macro	**81.39**	**92.40**	**86.54**	**64.16**	**92.92**	**74.90**

#### Main results

We compare RMP with FMP (Forward Maximum Matching algorithm with POS) in terms of constructing distant labels on Label 1 and Label 2. Table 4 presents the precision, recall, and F1 scores of both methods for two entity classes, along with the corresponding micro and macro metrics. On both labels, RMP outperformed FMP, indicating that RMP is indeed superior to FMP on this dataset. Specifically, (1) the overall performance of Label 1 is better than Label 2, particularly in terms of precision and F1 scores. However, in terms of recall, especially for the Product class, Label 2 exhibits higher recall than Label 1. This may be because the Product class has a broader scope, which Label 1 cannot fully cover, while Label 2, with its larger dictionary space, can encompass it effectively; (2) due to the larger dictionary space in Label 2, its precision significantly lags behind Label 1, introducing more noise. On the other hand, Label 1 maintains overall high quality. Therefore, high-confidence texts primarily rely on Label 1.

#### Ablation study

In order to further validate the effectiveness of the proposed POS condition filtering, we conduct ablation experiments. “**w/o POS**” denotes the condition where POS filtering is not applied. The results are shown in Table 5. It can be observed that, for both Label 1 and Label 2, removing the POS condition filtering led to a certain decrease in label quality. While the recall slightly increased due to the absence of limiting conditions, the significant decrease in precision and F1 scores outweighs this minor increase. These results demonstrate the necessity of adding POS condition restrictions to improve the quality of distant labels.

**Table 5:** Ablation experiment results (in %).

Ablations	Entity Class	Pre	Rec	F1
**Label 1**	Vendor	83.56	94.52	88.70
	Product	79.21	90.28	84.38
**w/o POS**	Vendor	81.71(-1.85)	95.83(+1.31)	88.21(-0.49)
	Product	68.81(-10.40)	90.56(+0.28)	78.20(-6.18)
**Label 2**	Vendor	76.33	88.80	82.09
	Product	51.99	97.05	67.71
**w/o POS**	Vendor	71.10(-5.23)	89.15(+0.35)	79.11(-2.98)
	Product	39.70(-12.29)	97.33(+0.28)	56.39(-11.32)

### Improved self-training experiment results

To validate the effectiveness of the proposed IST algorithm in mitigating the impact of noise in distant labels and improving the performance of NER models, we conduct experiments using various NER models that have been proven to perform well on NER tasks. We selected different embedding methods and network architectures for comparison.

#### Main results

Table 6 presents the performance comparison of all methods in terms of precision, recall, and F1 score. From the table, it can be observed that the performance of all models trained on distant labels is significantly inferior to that of models trained under full supervision with true labels. This indicates that the distant labels contain a substantial amount of noise, leading to a decrease in model performance. However, our proposed IST algorithm has greatly improved the metrics for all models compared to training directly on Label 1. Specifically, (1) all models show improvements in precision, recall, and F1 score. Notably, the proposed constrained self-training algorithm significantly enhanced the F1 scores of several state-of-the-art NER models on this dataset, achieving a 3.5% improvement for the Vendor class and a 3.35% improvement for the Product class. Among them, BiLSTM-CRF exhibits the largest improvement in F1 score for the Vendor class, with an increase of 4.35%, while the BiLSTM-CNN-CRF model demonstrates the largest improvement of 3.95% in the Product class. (2) The best-performing models among the five are BiLSTM-CNN-CRF, RoBERTa, and RoBERTa-wwm-RDCNN-CRF with RoBERTa-wwm-RDCNN-CRF’s IST algorithm producing results closest to those obtained with true label training. The results indicate that pre-trained models, due to their better generalization capabilities, can fit the true distribution more effectively during self-training, resulting in overall better performance. The experimental results demonstrate that our proposed IST algorithm effectively reduces the noise impact of distant labels and improves the performance of the NER models.

**Table 6:** Comparsion of NER results (in %).

Methods	Class	Distant Supervison					Full Supervision			
		Label 1				IST	Groud Truth			
		Pre.	Rec.	F1	Pre.	Rec.	F1	Pre.	Rec.	F1
BiLSTM-CRF	vendor	76.89	80.00	78.41	80.34	85.34	82.76	89.98	88.79	89.38
	product	71.65	79.88	75.54	75.55	82.09	78.68	88.23	86.34	87.27
LSTM-CNN	vendor	76.65	81.39	78.95	80.56	85.54	82.98	90.23	88.87	89.54
	product	72.07	79.44	75.58	75.43	82.37	78.75	88.89	86.44	87.65
BiLSTM-CNN-CRF	vendor	80.98	88.11	84.39	83.93	91.89	87.73	94.49	93.72	94.10
	product	74.80	85.22	79.67	79.45	88.25	83.62	90.33	90.00	90.16
RoBERTa	vendor	80.70	90.23	85.20	83.71	92.03	87.67	93.20	94.02	93.61
	product	75.21	87.28	80.80	80.56	88.28	84.24	91.09	90.88	90.98
RoBERTa-wwm-RDCNN-CRF	vendor	81.26	89.97	85.39	85.21	92.54	88.72	94.76	94.15	94.45
	product	76.69	88.14	82.02	81.36	89.17	85.09	91.28	90.32	90.80

#### Parameter study

**Fig 3:**
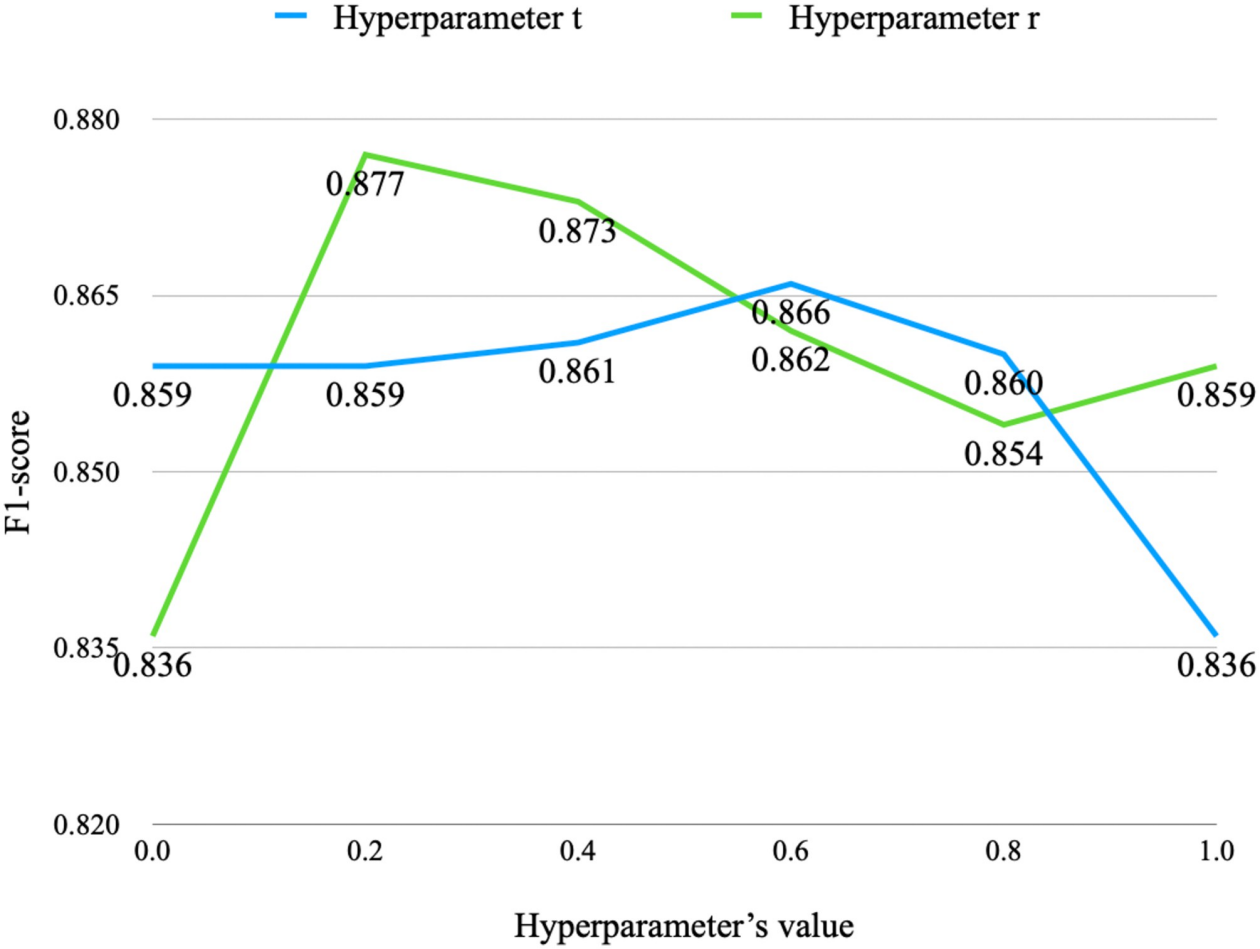
Influence of hyperparameters on experimental results in IST algorithm.

We explored the influence of two hyperparameters *t* and *r* on the experimental results, as shown in Eqs 7 and 8. Fig 3 presents the experimental results, where the blue curve represents the influence of hyperparameter *r*, and the red curve represents the influence of hyperparameter *t*. From the experimental analysis, we observed that the model is relatively insensitive to *t*, but the F1 peak achieved by it is lower than that of *r*. Additionally, *r* remains insensitive within a certain range to achieve the F1 peak. Therefore, for actual model training and application, we chose *r* to constrain the value of *C*_*update*_, and the selected value for *r* is 0.2.

## Conclusion and future work

In this study, we investigate the problem of distant supervision for NER in the field of cybersecurity. To obtain the distant labels, we first create two cybersecurity domain dictionaries, then we propose a method that combines reverse maximum matching and POS tag filtering to construct a distant dataset. To mitigate the noise introduced by distant labels and improve the performance of NER models, we introduce an improved self-training algorithm. This algorithm first trains the NER model on high-confidence texts and then updates the confidence weights through constraint conditions using a teacher-student model for self-training. Our approach effectively alleviates the noise impact of distant labels and significantly improves the performance of multiple state-of-the-art NER models on this distant dataset.

While our proposed method demonstrates significant potential, it is important to highlight some limitations and potential areas for future research. Firstly, we only utilized two cybersecurity lexicons for constructing the distant labels, which may limit the coverage of domain-specific terms. In future work, we could expand our approach by incorporating additional and more comprehensive cybersecurity dictionaries. Secondly, the IST algorithm proposed in this study relies heavily on the quality and quantity of annotated data. This can pose challenges in scenarios where labeled data is scarce. To address this limitation, future research could explore the integration of meta-learning and other low-resource domain training techniques with natural language processing. Such approaches could guide the selection of labeled data more effectively and tackle the issue of limited resources in small-sample scenarios.
